# Women’s alcohol use in mid-life: Identifying associations between menopause symptoms, drinking behaviour, and mental health

**DOI:** 10.1177/17455057251359767

**Published:** 2025-10-08

**Authors:** Emma L. Davies, Sam Burton, Rebecca Monk, Elena Murdoch, Eiluned Pearce, Abigail K. Rose

**Affiliations:** 1 Centre for Psychological Research, Oxford Brookes University, UK; 2 School of Psychology, Liverpool John Moores University, UK; 3 Department of Women’s and Children’s Health, Faculty of Life Sciences & Medicine, School of Life Course and Population Sciences, King’s College London, UK; 4 Department of Psychology, Edge Hill University, Ormskirk, UK; 5 Division of Psychiatry, University College London, UK

**Keywords:** alcohol, menopause, women’s health, mental health, drinking motives, negative reinforcement

## Abstract

**Objectives:**

The study aimed to identify associations between menopause, alcohol use, and mental health. Particularly examining how menopause symptoms may relate to alcohol behaviours in midlife women.

**Design:**

An online mixed methods cross-sectional survey study was conducted to gather data on menopause-related symptoms, alcohol use, mental health, and well-being among women.

**Methods:**

A sample of 936 women aged 40–65 was recruited for the study. Quantitative measures assessed menopause symptoms, alcohol use and associated harm, mental health, and overall well-being. Qualitative data were collected through text responses to explore motives for drinking.

**Results:**

Perimenopausal participants, compared to pre- or postmenopausal women, reported the highest levels of menopause symptoms, negative reinforcement drinking motives (e.g. drinking to cope), negative mood, and the lowest well-being scores. Additionally, negative reinforcement motives partially mediated the relationship between menopausal symptoms and hazardous drinking. Qualitative findings showed that women often drank as a coping mechanism, while some avoided alcohol due to its potential to worsen menopausal symptoms.

**Conclusions:**

This study provides new insights into the associations between menopausal symptoms, alcohol use, and mental health in midlife women. The findings highlight the complex factors driving alcohol use and avoidance, suggesting that tailored interventions are needed for women in midlife. This research underscores the importance of addressing alcohol-related risks in this under-researched group, given the potential for significant harm.

## Introduction

There are sex differences in patterns of alcohol use, consequences of drinking, and treatment outcomes.^1^ During midlife, when most women experience stages of menopause, there are several social, hormonal, and biological processes, which may influence alcohol use, and increase risk of alcohol harm and mental ill-health.^2^ However, there is very little research focusing on the intersection of women’s drinking, mental health, and menopausal symptoms.

Natural menopause typically starts around 49 years with a range between 46 and 52 years.^3^ Using the Stages of Reproduction Aging Workshop + 10 system, ‘perimenopause’ covers the early and late menopausal transition phases, with the 12 months following the last period retrospectively labelled as ‘menopause’.^4^ Following this, women are considered postmenopausal for the rest of their lives. These stages are associated with several criteria around cycle length and levels of follicle-stimulating hormone and oestradiol (oestrogen).^4^

During perimenopause/menopause, females may notice a range of physical (e.g. atypical menstrual cycles, hot flashes, night sweats, joint pain, weight gain, hair loss, vaginal dryness/itching/pain), cognitive (e.g. sleep disturbance, cognitive impairments), psychological and emotional (e.g. reduced libido, mood swings, low mood, depression, anxiety, suicidal ideation) symptoms.^5,6^ These symptoms can influence one another and can continue for decades into postmenopause, for instance one third of women report vasomotor symptoms up to 79 years of age.^7^ During stages of menopause, women are also more at risk of experiencing mental ill-health (e.g. depression and anxiety) compared with premenopausal females.^6,8^ This wide and often debilitating range of symptoms can make it difficult to cope with everyday life.^6^ Given the potential longevity of these symptoms, it is important to understand the impact stages of menopause may have on other key health behaviours.

Complex changes in sex hormone activity and levels occur during perimenopause, including erratic oestrogen activity before an eventual decline in oestrogen and progesterone levels.^6^ Sex hormones influence various neurochemical regulatory systems, including those responsible for mood, sleep, and behaviour,^9^ likely contributing to negative menopausal symptoms. It is well-established that negative moods (e.g. anxiety, depression, loneliness, stress) can be associated with higher alcohol use and harm,^10^ and adult women report using alcohol to cope with negative states,^11^ with some research suggesting that women (relative to men) are more likely to use alcohol for coping based reasons.^12^ Therefore, during menopause, a heightened risk of hormone-related declines in physical and mental health may drive elevated alcohol use to cope with negative menopausal symptoms.^2^ According to the self-medication hypothesis, drinking can be driven by negative reinforcement motives such as the desire to reduce/remove aversive states.^13^ As such, hormone fluctuations during menopause may be an important consideration in understanding alcohol consumption. Indeed, research suggests that women who did not previously engage in excessive drinking are more likely to transition to excessive drinking as they progress through the stages of the menopausal transition,^14^ suggesting there are unique elements associated with this life stage that may confer risk for increased alcohol consumption.

In addition to elevated negative reinforcement value of alcohol, owing to (peri)menopause-related neurochemical changes, oestrogen and progesterone can determine the level of positive reward from consumption. Specifically, drinking during menopause is associated with increased oestrogen levels^15^ and both oestrogen and progesterone can influence neurotransmitters involved in determining alcohol behaviour. Higher levels of oestrogen can enhance motivation to consume alcohol in animal and human females,^16,17^ while progesterone may counteract these effects, although there is some inconsistency in the evidence.^18–20^ This means that alcohol’s impact on hormones may be an important part of understanding drinking during menopause transition and beyond.

Additionally, age-related declines in lean muscle mass, increased body fat ratio, and lower levels of alcohol dehydrogenase (enzyme primarily responsible for breaking down alcohol) reduce women’s ability to metabolise alcohol as they age.^20,21^ This contributes to increased sensitivity to alcohol and vulnerability to alcohol-related toxicity and other harms.^2,20^ Overall, the relationship between menopause and alcohol consumption likely involves complex interactions between hormone fluctuations, physiology changes and the impact of consumption on the body, and the impact this has on the harmful and rewarding effects of drinking.

Yet it is important to remember that mental ill health is associated with higher rates of hazardous and harmful drinking in some and higher rates of abstinence in others.^22,23^ This suggests mental ill-health can have a polarising effect on alcohol use. Thus, when considering women experiencing perimenopause/menopause, a self-medication framework may be an overly homogenising representation of consumption. Indeed, the evidence suggests several pathways by which alcohol use and menopause symptoms may influence each other. The aim of this novel study was therefore to determine associations between menopause and drinking behaviour and identify predictors of increased alcohol use. We hypothesised that women experiencing more severe menopausal symptoms and mental ill health would report higher levels of alcohol use and endorse negative reinforcing drinking motives.

## Methods

The reporting of this study conforms to the STROBE statement.^24^

Study design and setting.

An online mixed methods cross-sectional survey study.

### Participants

To capture pre–post-menopause stages, participants were aged 40–65 years. Inclusion criteria for the study were being assigned female at birth, and aged 40–65. Exclusion criteria were being outside of this age range, assigned male at birth, not being fluent in English, or currently being pregnant (due to UK recommendations of abstinence during pregnancy).

### Measures

The full survey can be seen in Supplementary File A.

*Demographics*: Age, ethnicity, sexuality, gender identity, relationship status, highest level of education, current occupation, average household income (before tax), number and age of children, UK area of residence.

*Menopause status and menstruation*: Participants self-identified as premenopausal, peri/menopausal, or postmenopausal (yes, no, not sure). Each status included a brief definition of the status and possible symptoms (or lack of), and the full description of each status is shown in Supplementary File A. They were also asked whether they were taking medication to manage menopause symptoms (yes, no, not sure); what medications were being taken (e.g. oestrogen, progesterone, anti-depressants); age symptoms first noticed; cause of menopausal symptoms (e.g. natural, cancer, gynaecological surgery, other); date of first day of last menstruation; occurrence of full/partial hysterectomy.

*Menopause symptoms*: The Menopause-Specific Quality of Life (MENQOL) Questionnaire^25^ assessed the presence and impact of menopause symptoms (29 items; α = 0.949) using a Likert scale. Items fall within one of four symptom domains: vasomotor (3 items; α = 0.865), psychosocial (7 items; α = 0.905), physical (16 items; α = 0.925), sexual (3 items; α = 0.820). Participants can rate the degree to which the individual is bothered by each symptom (0 = not bothered at all, 6 = extremely bothered), and then subscales are summed.

*Historical alcohol consumption*: Participants retrospectively reported the type and quantity of alcoholic drink they consumed p/week prior to noticing symptoms of menopause. Drinks were converted to units (1 UK unit = 8 g alcohol) to calculate weekly alcohol unit consumption.

*Current alcohol consumption*: The Timeline Followback assessed weekly alcohol use.^26^ Using a diary format, participants were asked to record how many and what type of drink (e.g. large/small glass of wine, pint of beer) they had consumed over the past 14 days. Drinks were converted to units and an average was calculated for weekly consumption.

*Alcohol harm*: The Alcohol Use Disorders Identification Test (AUDIT) assessed alcohol use and potentially harmful drinking behaviour (10 items; α = 0.833) with the Alcohol Use Disorders Identification Test - Consumption (AUDIT-C) comprising the first three items which represent current consumption (three items; α = 0.769).^27^ Scores indicate 0–7: low-risk drinking, 8–15: increasing risk, 16–19: higher risk, 20+ possible dependence. For women, it is recommended that low-risk drinking is scored 0–4^28^, this is included in Table 1. When using the term ‘AUDIT score’ in the remainder of article, we are referring to the total score out of 40.

**Table 1.** Drinking behaviours, drinking motives, and mental health.

	Whole sample	Premenopausal	Perimenopausal	Postmenopausal	Not sure	p value *
Drinker status, N = 936		N = 115	N = 387	N = 323	N = 111	
Non-drinker, N (%)	107 (11.5)	16 (13.9)	37 (9.6)	42 (13.0)	12 (10.9)	0.413
Current drinker, N (%)	829 (88.5)	99 (86.1)	350 (90.4)	281 (87.0)	99 (89.1)	
AUDIT categories drinkers, N = 827						
Low risk, N (%) (0–7)	571 (69.0)	66 (66.7)	223 (63.9)	216 (76.9)	66 (67.3)	0.045
Increasing risk, N (%) (8–15)	196 (23.7)	26 (26.3)	95 (27.2)	48 (17.1)	27 (27.6)	
Higher risk, N (%) (16–19)	29 (3.5)	3 (3.0)	18 (5.2)	7 (2.5)	1 (1)	
Possible dependence, N (%) (20+)	31 (3.7)	4 (4.0)	13 (3.7)	10 (3.6)	4 (4.1)	
Gendered AUDIT cut off, N = 827						
Low risk (0–4)	397 (48)	42 (42.4)	153 (43.8)	156 (55.5)	46 (46.5)	0.018
Hazardous (5+)	430 (52)	57 (57.6)	196 (56.2)	125 (44.5)	52 (53.1)	
Alcohol unit risk groups						
0–14 units, N (%)	556 (68.8)	69 (71.1)	225 (66.8)	193 (69.9)	69 (70.4)	0.933
15–34 units, N (%)	177 (21.9)	21 (21.6)	80 (23.7)	56 (20.3)	20 (20.4)	
35 units and above, N (%)	75 (9.3)	7 (7.2)	32 (9.1)	27 (9.6)	9 (9.2)	
Alcohol consumption (units)						
Units pre-menopause, M (SD)	18.49 (25.23)	–	18.47 (24.96)	19.54 (26.07)	15.56 (23.57)	0.359
Units pre-menopause, median (IQR)	10 (22.23)		9.9 (22.55)	11.2 (22.55)	7.7 (20.45)	
Current weekly units, M (SD)	13.48 (17.88)	11.59 (11.90)	14.16 (17.69)	12.95 (16.96)	14.48 (24.80)	0.539
Current weekly units, median (IQR)	7.45 (16.39)	7.25 (16.64)	8.4 (17.73)	6.6 (16.29)	6.13 (14.16)	
AUDIT score, M (SD)	6.37 (5.33)	6.53 (5.20)	6.92 (5.55)	5.57 (5.02)	6.53 (5.30)	0.016
AUDIT score, median (IQR)	5 (6)	6 (7)	5 (7)	4 (5)	5 (5.25)	
AUDIT-C, M (SD)	4.52 (2.71)	4.61 (2.64)	4.79 (2.80)	4.14 (2.59)	4.49 (2.74)	0.026
AUDIT-C, median (IQR)	4 (4)	4 (5)	4 (5)	4 (3)	4 (4)	
Drinking motives						
Negative motives	18.37 (8.71)	16.46 (7.14)	19.24 (8.97)	17.81 (8.76)	18.59 (8.69)	0.030
Positive motives	14.12 (4.19)	14.36 (4.38)	14.15 (3.98)	13.71 (4.44)	14.93 (3.95)	0.092
Whole sample						
Mental health, M (SD)						
MENQOL	2.48 (1.29)	1.89 (1.31)	2.72 (1.21)	2.45 (1.29)	2.32 (1.29)	<0.000
Loneliness	5.03 (2.00)	5.05 (2.06)	5.14 (2.01)	4.84 (1.95)	5.14 (2.02)	0.231
DASS	5.59 (5.54)	4.86 (4.86)	6.47 (5.97)	4.97 (5.21)	5.39 (5.21)	<0.001
WHO-5	44.90 (22.65)	49.86 (21.17)	41.12 (21.85)	48.24 (23.10)	43.20 (23.44)	<0.000

Chi square p value for drinker status, unit consumption and AUDIT categories and one-way ANOVA p value for other measures.

AUDIT: Alcohol Use Disorders Identification Test; DASS: Depression, Anxiety, and Stress Scale; IQR: Interquartile Range; MENQOL: Menopause-Specific Quality of Life; WHO-5: World Health Organisation’s Wellbeing Scale.

*Drinking motives*: The Maternal Drinking Motive Scale (MDMS) assessed drinking motives.^29^ The MDMS is based on the well-validated Drinking Motives Questionnaire which was developed with adolescents.^30^ The MDMS includes items more appropriate for older, female respondents. Instead of asking participants ‘since being a mother how often have you drunk alcohol because. . .’, in the current study, participants were asked ‘How often do you drink alcohol. . .’. Items fall across two domains: positive reinforcement motives (e.g. to celebrate, to have fun, five items; α = 0.822) and negative reinforcement motives (e.g. to cope with stress, to reduce low mood, eight items; α = 0.937).

*Motives to reduce/stop drinking*: There are no specific scales measuring this topic. Therefore, we included 14 items selected based on existing evidence (e.g. to be healthy, to manage anxiety, to sleep better).

*Qualitative responses*: Given the lack of research in this area, following the motives to drink and not to drink items, respondents were given the opportunity to provide free text responses. These were provided to highlight and expand on their motives to drink and to reduce drinking, in addition to commenting on how they believed their reasons to drink had changed since noticing menopause symptoms.

*Mental health*: The Depression, Anxiety, and Stress Scale-8 (DASS-8, eight items; α = 0.906) assessed negative affect across anxiety (three items), depression (three items), and stress (two items) using a Likert-type scale to 0 (‘Did not apply to me at all’) to 3 (‘Applied to me very much, or most of the time’).^31^ A mean score for each subscale was calculated.

*Loneliness*: The UCLA Loneliness Scale assessed loneliness over three items (α = 0.898), scored: 1 = hardly ever, 2 = some of the time, and 3 = often.^32^ A total score is calculated with higher scores indicating greater loneliness.

*Well-being*: The World Health Organisation’s Wellbeing Scale (WHO-5) assessed current well-being with five items (α = 0.895) scored: 0 = at no time to 5 = all of the time.^33^ A total score is computed by summing responses and multiplying by 4 to give a percentage. Items are positively framed (e.g. I have felt cheerful and in good spirits).

### Procedure

The online survey was advertised through social media, community sites, and Prolific (an online research recruitment platform) to increase diversity in participant pool.^34^ Participants provided written informed consent online before the survey was launched (ethical approval was obtained from Liverpool John Moores University, PSY-Rec (code: 22-PSY-020)). The survey presented questions/scales following the order above. Each section provided an overview of the topics to be covered and a reminder that responses were anonymous and that questions were asked with compassion and without judgement. An attention check was included (What planet do you live on?). Links to alcohol and menopause support sources were provided at the end of the survey. The survey took ~20 min to complete and ran from January 2023 to June 2023.

### Analysis

Missing data were dealt with using pairwise deletion given the small number of missing items, once the data were cleaned by excluding unsubmitted/suspected careless responses (n = 44). Descriptive statistics were used to explore the sample characteristics, including menopause status, drinking variables, and mental health variables. We compared respondents at different stages of the menopause using ANOVAs and chi-squared tests to explore associations between variables. We similarly compared the study measures between respondents using Hormone Replacement Therapy (HRT) or not (see Supplementary Materials). While some measures were skewed, the sample size was considered large enough for parametric tests to be appropriate.^35^ We used correlations to examine relationships between study measures for respondents who identified as current drinkers.

We compared low risk (AUDIT score <8) and hazardous/higher risk drinkers (AUDIT ⩾8) on MENQOL, negative affect, and drinking motives for each stage of the menopause (while controlling for HRT) and include this as a Supplementary Table 2. It is important to note that this number of multiple comparisons increases the chance of a type 1 error and so these findings should be used to indicate possible trends for further exploration.

A Negative Binomial regression model was used, given significant over-dispersion based on residuals and non-normally distributed data, to explore predictors of AUDIT score (for people who were current drinkers). AUDIT score was entered as the count outcome variable. Menopause status, HRT use, ethnicity, and education were entered as factors and MENQOL subscales, loneliness, DASS, WHO-5, drinking motives, and income were entered as co-variates (a similar model with DASS as the outcome variable is shown in Supplementary Table 3). Finally, we used the PROCESS tool in SPSS (SPSS 27, IBM)^36^ to explore whether drinking motives mediated the relationship between MENQOL and AUDIT score, adjusting for menopause and HRT status (see Supplemental Table 3 for regression and mediation analysis for the mental health outcome (DASS)).

For qualitative data, we employed an ‘exploratory descriptive approach’, where data is described to provide a better understanding of an aspect of social/psychological life and is particularly useful for new areas of research.^37^ This theoretical method can guide coding across a priori topics while following the six phases of thematic analysis.^38^ The authors independently familiarised themselves with the data, and initial codes were generated independently. A group discussion with authors finalised themes and subthemes, which helped establish the trustworthiness of the data.^39^ The three a priori topics were: ‘motives to drink’, ‘motives to reduce drinking’, and ‘changes in motives since noticing perimenopausal symptoms’. Due to the brevity of responses, we purposefully avoided potential over-interpretation of the data and generated semantic themes with the aim of guiding future research.^40^

## Results

### Sample characteristics

One thousand and eighteen participants completed the survey and the final sample consisted of 936 respondents, after removing participants who did not pass the attention check, did not meet inclusion criteria, or who provided inconsistent patterns of responding. Nine hundred and twenty identified as women, four were non-binary, four selected ‘other’, and one identified as a trans man. A further seven chose not to respond to this question. Other characteristics of the sample are shown in Table 2. The average age in the sample was 50.71 (SD = 6.86). One hundred and fifteen (12.3%) of the respondents were premenopausal, 387 (41.3%) were perimenopausal, 323 (34.5%) were postmenopausal, and 111 (11.9%) were unsure. The average age of those who selected ‘not sure’ was in line with those who were perimenopausal, and may reflect that those respondents had experienced some menopausal symptoms and had not sought medical advice, or were not sure whether their symptoms were related to menopause. Three quarters of the respondents (75%) were not currently using HRT. The sample was predominantly White British with most educated to degree level or above (see Table 2 for full characteristics and Supplemental Table 1 for characteristics by HRT status).

**Table 2.** Sample characteristics.

Characteristic	Whole sample	Premenopausal	Perimenopausal	Postmenopausal	Not sure
Total, N (%)	936 (100)	115 (12.3)	387 (41.3)	323 (34.5)	111 (11.9)
HRT status, N (%)					
On HRT	234 (25)	19 (16.5)	113 (29.2)	86 (26.6)	16 (14.4)
Not on HRT	702 (75)	96 (83.5)	274 (70.8)	237 (73.4)	95 (85.6)
Age, mean (SD)	50.71 (6.86)	44.85 (4.70)	47.94 (4.40)	57.35 (4.82)	47.13 (5.95)
Ethnicity, N (%)					
Asian—British	38 (4.1)	4 (3.5)	22 (5.7)	7 (2.2)	5 (4.5)
Asian—other	7 (0.7)	2 (1.7)	2 (0.5)	1 (0.3)	2 (1.8)
Black—British	43 (4.6)	8 (7.0)	21 (5.4)	11 (3.4)	3 (2.7)
Black—other	8 (0.9)	1 (0.9)	1 (0.3)	4 (1.2)	2 (1.8)
Mixed—any	55 (5.9)	16 (13.9)	20 (5.2)	10 (3.1)	9 (8.1)
White—British	715 (76.4)	77 (67.0)	292 (75.5)	266 (82.4)	80 (72.1)
White—other	61 (6.5)	7 (6.1)	25 (6.5)	20 (6.2)	9 (8.1)
Any other	5 (0.5)	–	2 (0.5)	3 (0.9)	–
Prefer not to say	4 (0.4)	–	2 (0.5)	1 (0.3)	1 (0.9)
Education					
GCSE or below	99 (10.6)	8 (7.0)	30 (7.8)	46 (14.3)	15 (13.5)
A-level	129 (13.8)	12 (10.4)	49 (12.7)	50 (15.5)	18 (16.2)
Technical	125 (13.4)	13 (11.3)	46 (11.9)	53 (16.5)	13 (11.7)
Batchelor’s degree	333 (35.6)	43 (37.4)	149 (38.5)	107 (33.2)	34 (30.6)
Master’s	130 (13.9)	18 (15.7)	53 (13.7)	39 (12.1)	20 (18.0)
Doctorate	63 (6.7)	12 (10.4)	32 (8.3)	11 (3.4)	8 (7.2)
Professional degree	41 (4.4)	9 (7.8)	20 (5.2)	12 (3.7)	–
Other	16 (1.7)	–	8 (2.1)	5 (1.5)	3 (2.7)
Occupation, N (%)					
Full-time employment	370 (39.6)	60 (52.5)	176 (45.5)	87 (26.9)	47 (42.3)
Part-time employment	226 (24.2)	23 (20)	94 (24.3)	85 (26.3)	24 (21.6)
Self-employed	117 (12.5)	16 (13.9)	51 (13.2)	39 (12.1)	11 (9.9)
Homemaker	88 (9.4)	6 (5.2)	40 (10.4)	28 (8.7)	14 (12.6)
Retired	61 (6.5)	2 (1.7)	2 (0.5)	56 (17.3)	1 (0.9)
Unable to work	36 (3.9)	2 (1.7)	11 (2.8)	17 (5.3)	6 (5.4)
Unemployed	26 (2.8)	5 (4.3)	8 (2.1)	8 (2.5)	5 (4.5)
Other	12 (1.2)	1 (0.9)	4 (1.3)	3 (0.9)	3 (2.7)
Household income, median (IQR)	£45,000	£54,000	£51,000	£36,000	£42,000

GCSE: General Certificate of Secondary Education.

### Sample drinking behaviours and mental health

In the sample, 107 (11.5%) were non-drinkers, and 829 (88.5%) were current consumers of alcohol (see Table 1). On average, respondents consumed a median of 7.45 units of alcohol each week. Using standard AUDIT scoring, the majority were classified as low risk drinkers (median: 6), but using the lower gender-based recommended AUDIT scores, most of the sample were classified as hazardous drinkers. Respondents in perimenopause had the highest mean score for negative drinking motives (MDMS: 19.24), menopause symptoms (MENQOL: 2.72), and negative affect (DASS: 6.47), and the lowest mean score on well-being (WHO-5: 41.12).

### Correlations between alcohol, mental health, and menopause measures for current drinkers

Higher AUDIT score was positively associated with higher levels of menopause symptoms (MENQOL total and all domains), negative affect (DASS), and loneliness, lower well-being (i.e. WHO-5), and greater endorsement of both negative and positive reinforcement drinking motives, *ps* <0.01. Higher weekly consumption was also associated with these outcome measures, *ps* <0.01, except for loneliness (see Table 3).

**Table 3.** Correlations between study measures for those respondents who currently drink alcohol.

Outcome measure	Average weekly units	AUDIT	AUDIT-C	MENQOL	MENQOL vasomotor	MENQOL psychosocial	MENQOL physical	MENQOL sexual	Loneliness	DASS	WHO-5	Negative motives	Positive motives
Before menopause units	0.520**	0.433	0.449**	0.097**	0.035	0.086*	0.085*	0.101**	0.067	0.123**	−0.077*	0.257**	0.151**
Average weekly units		0.711**	0.733**	0.120**	0.113**	0.118**	0.098**	0.079*	0.057	0.160**	−0.123**	0.382**	0.228**
AUDIT			0.870**	0.188**	0.103**	0.225**	0.159**	0.086*	0.165**	0.265**	−0.208**	0.476**	0.283**
AUDIT-C				0.121**	0.109**	0.154**	0.087*	0.057	0.056	0.138**	−0.136**	0.462**	0.338**
MENQOL					0.604**	0.862**	0.959**	0.642**	0.296**	0.509**	−0.514**	0.202**	0.027
MENQOL vasomotor						0.387**	0.509**	0.349**	0.037	0.157**	−0.165**	0.084*	0.027
MENQOL psychosocial							0.749**	0.456**	0.400**	0.646**	−0.615**	0.229**	0.031
MENQOL physical								0.524**	0.256**	0.438**	−0.464**	0.167**	0.009
MENQOL sexual									0.136**	0.244**	−0.223**	0.157**	0.054
Loneliness										0.524**	−0.470**	0.186**	0.035
DASS											−0.644**	0.287**	0.022
WHO-5												−0.255**	0.026
Negative motives													0.443**

AUDIT: Alcohol Use Disorders Identification Test; DASS: Depression, Anxiety, and Stress Scale; MENQOL: Menopause-Specific Quality of Life; WHO-5: World Health Organisation’s Wellbeing Scale.

* p < 0.05. **p < 0.01.

Menopause symptoms were positively associated with mental ill-health, loneliness, and endorsement of negative (but not positive) reinforcement drinking motives.

Negative drinking motives were more strongly associated with AUDIT scores and weekly unit consumption than positive motives (Fishers *z* for AUDIT = 4.53, p < 0.001; Fishers *z* for weekly consumption = 3.40, p < 0.001). None of the relationships indicated concerns about multicollinearity.

In addition, Supplementary Table 3 (controlling for HRT) shows relationships by drinking status (low risk: AUDIT score <8, hazardous/higher risk drinkers: AUDIT ⩾8). In participants who were pre-menopausal, hazardous drinkers had higher MENQOL scores, specifically in the psychosocial (*F* (1, 96) = 5.64, p < 0.05) and physical domains (*F* (1, 96) = 5.95, p < 0.05), and higher negative drinking motives (*F* (1, 88) = 19.39), p < 0.001). In perimenopausal participants, higher risk drinkers had higher scores on the MENQOL psychosocial domain (*F* (1, 346) = 19.12, p < 0.01), loneliness (*F* (1, 346) = 53.41, p < 0.001), WHO (*F* (1, 345) = 468.78, p *<* 0.01), DASS (*F* (1, 338) = 530.96, p < 0.001), and higher negative (*F* (1, 337) = 3567.61, p < 0.001) and positive drinking motive scores (*F* (1, 341)) = 347.90, p < 0.001). However, for post-menopausal participants, the only difference was that hazardous drinkers endorsed negative (*F* (1, 267) = 3061.81, p < 0.001) and positive drinking motives (*F* (1, 269)) = 434.93, p *<* 0.001) more than low-risk drinkers.

### Predictors of hazardous drinking

The negative binomial regression model (see Table 4) was a significant fit compared to an intercept only model *χ*^2^ (20) = 142.54, p < 0.001. Menopause, MENQOL domains, DASS, loneliness, HRT status, education, ethnicity, and income were not significant predictors of AUDIT score in the model. Negative drinking motives (incidence rate ratio (IRR) = 1.043, 95% CI, 1.032–1.055), and positive drinking motives (IRR = 1.034, 95% CI, 1.012–1.056) were significant predictors of higher AUDIT score.

**Table 4.** Negative binomial regression model showing variables associated with AUDIT scores for participants who were current drinkers (i.e. AUDIT >0) in the sample.

	*B*	Standard error	Wald χ^2^	Sig.	IRR	95% CI	
						Lower	Upper
Intercept	0.403	0.357	1.274	0.259	1.496	0.743	3.011
Menopause							
Not sure	−0.084	0.168	0.25	0.617	0.919	0.662	1.278
Post-menopausal	−0.161	0.143	1.268	0.26	0.851	0.644	1.126
Peri-menopausal	−0.04	0.136	0.085	0.77	0.961	0.737	1.254
HRT							
Using HRT	0.051	0.098	0.268	0.605	1.052	0.868	1.275
**Education**							
Postgraduate	−0.012	0.117	0.011	0.918	0.988	0.785	1.243
Degree	−0.048	0.097	0.251	0.616	0.953	0.788	1.152
Ethnicity							
Other	0.163	0.506	0.103	0.748	1.177	0.436	3.172
White	0.215	0.216	0.993	0.319	1.24	0.812	1.894
Mixed	0.154	0.268	0.329	0.566	1.166	0.69	1.971
Black	−0.226	0.278	0.661	0.416	0.798	0.462	1.376
MENQOL							
Vasomotor	0.021	0.028	0.598	0.439	1.022	0.968	1.078
Psychosocial	0.027	0.047	0.331	0.565	1.027	0.937	1.127
Physical	−0.013	0.051	0.068	0.794	0.987	0.893	1.09
Sexual	−0.019	0.026	0.561	0.454	0.981	0.933	1.032
Mental health							
Loneliness	0.003	0.024	0.016	0.898	1.003	0.958	1.05
DASS	0.01	0.011	0.71	0.399	1.01	0.987	1.032
WHO-5	−0.002	0.003	0.394	0.530	0.998	0.994	1.003
Drink motives							
Negative	0.042	0.006	52.773	<0.001	1.043	1.032	1.055
Positive	0.033	0.011	9.583	0.002	1.034	1.012	1.056
Income	0	0.001	0.032	0.859	1	0.998	1.002

N = 757; probability distribution = negative binomial; link function = log; goodness of fit value/*df* = 0.401; omnibus test *χ*^2^ = 142.54 (20), p < 0.001 (see Supplemental Table 3 for regression analysis identifying MENQOL’s psychosocial domain, loneliness, and WHO-5 as significant predictors of mental ill-health (DASS)).

AUDIT: Alcohol Use Disorders Identification Test; DASS: Depression, Anxiety, and Stress Scale; IRR: incidence rate ratio; MENQOL: Menopause-Specific Quality of Life; WHO-5: World Health Organisation’s Wellbeing Scale.

### Mechanisms of association between menopause symptoms and hazardous drinking

**Figure 1.**
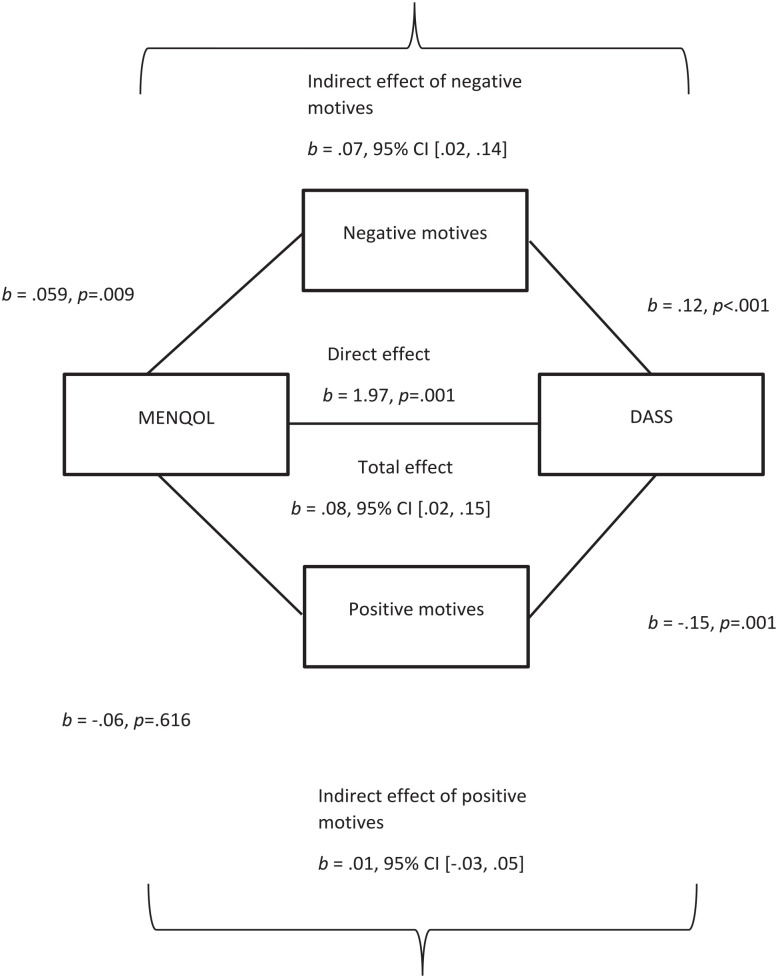
Multiple mediation model of the relationship between MENQOL and DASS, mediated by negative and positive drinking motives. Co-variates in the model are AUDIT, menopause status and HRT status. The confidence interval for the indirect effect is a BCa (bias-corrected and accelerated) bootstrapped CI based on 5000 samples, *R*^2^ = 0.306, p < 0.001. AUDIT: Alcohol Use Disorders Identification Test; DASS: Depression, Anxiety, and Stress Scale; MENQOL: Menopause-Specific Quality of Life.

MENQOL domains were significantly correlated with alcohol measures and DASS in bivariate analyses but failed to contribute to the regression model predicting AUDIT score. To explore the impact of MENQOL (total score) on AUDIT score, we constructed a multiple mediation model with positive and negative drinking motives as mediators, adjusting for menopause and HRT status (see Table 5). Negative reinforcement drinking motives (but not positive) significantly partially mediated the relationship between MENQOL and AUDIT score (see Figure 1), in that MENQOL was associated with increased negative drinking motives, which in turn were associated with increased AUDIT score.

**Table 5.** Bootstrapped standardised indirect effects for multiple mediation models to test whether drinking motives mediate the relationship between MENQOL and AUDIT score for respondents who currently drink alcohol.

	AUDIT, N = 788	95% CI^a^
Total	0.3021	0.1490–0.4771
Indirect effect of positive motives	0.0128	−0.0180 to 0.0531
Indirect effect of negative motives	**0.2893** ^b^	**0.1502–0.4524**

AUDIT: Alcohol Use Disorders Identification Test; MENQOL: Menopause-Specific Quality of Life.

a Bootstrapping confidence intervals based on 5000 samples.

b Significant mediation effect; covariates in the AUDIT model were menopause status and HRT status.

Bold values indicate Statistical significance.

See Supplemental Table 3 and Figure 1 for a mediation model demonstrating that negative reinforcement drinking motives mediated the relationship between MENQOL and negative affect (DASS).

### Reasons for reducing alcohol consumption since noticing menopausal symptoms

**Figure 2.**
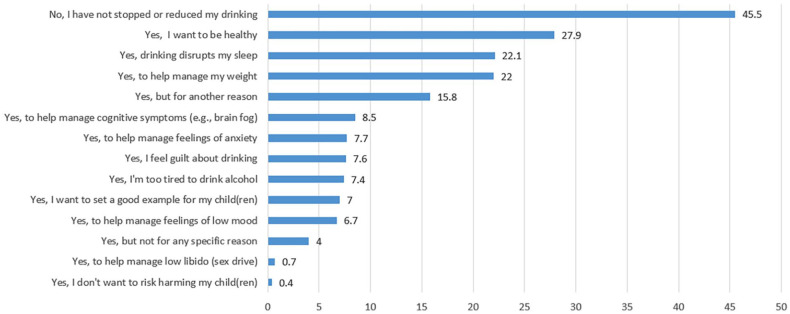
Since noticing symptoms associated with the stages of menopause, have you stopped or reduced your alcohol consumption for a specific reason? Data included for people identifying as peri- or post-menopausal only (N = 673).

The modal response was not stopping or reducing alcohol use since noticing symptoms associated with (peri)menopause (N = 306/673; 45.5%). Of those who did report a reduction in drinking, the most frequently cited motives were a desire to be healthy (N = 188; 27.9%) or to manage weight (N = 148; 22.0%) or sleep issues (N = 149; 22.1%). Less frequent responses were to avoid harm to children (N = 6; 0.6%) and to help manage libido (N = 6; 0.6%; see Figure 2).

### Qualitative results

**Figure 3.**
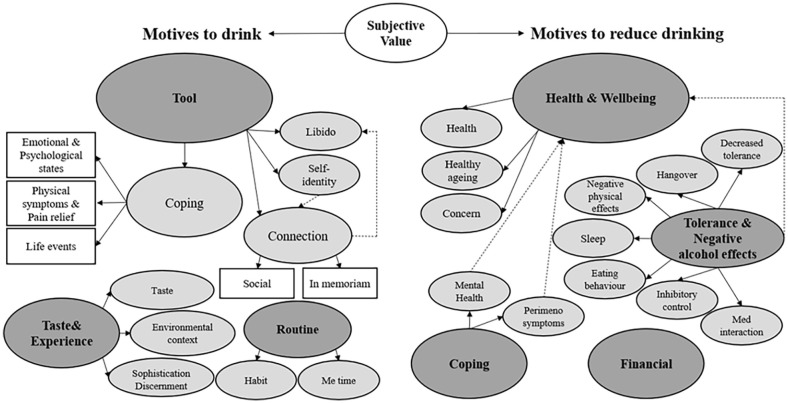
Schematic of qualitative themes. Women reported a range of motives to drink and to reduce drinking. Coping was a dominant theme across all a priori topics. Alcohol was used to manage negative physical and mental health symptoms. Although women reported reducing or stopping drinking to cope, the primary reasons for changing motives (since noticing perimenopausal symptoms) identified a shift from drinking for social reasons to drinking to cope both with health-related symptoms and life events. This highlights that (peri)menopause may be a stage of life where women are at risk of using alcohol either to self-medicate against various unwanted issues (e.g. mental/physical health symptoms, isolation) or to reward themselves for dealing with these issues. There was an awareness of ageing and how drinking behaviours could be detrimental but also used to shape self-identity. Women’s desire to be healthy highlights opportunities for health promotion, but the reported use of alcohol as a coping mechanism and pain reliever suggests opportunities are being missed. Dark grey: overarching themes; light grey: subthemes, level 1; white rectangle: subthemes, level 2.

Approximately 14%–16% of participants provided at least one free text response concerning the a priori topics: motives to drink, motives to reduce drinking, and changing motives since noticing menopausal symptoms. Within these topics, themes are shown underlined and subthemes in italics (see Figure 3 for themes/subthemes for motives to drink and motives not to drink).

Across all a priori topics, qualitative responses can be placed within a ‘subjective value-based’ framework (see “Discussion” section); with alcohol’s positive value relating to motives to drink (e.g. from helping someone to cope or enjoy themselves) and alcohol’s negative value relating to motives to not drink (e.g. impact on health and finances). Additionally, many of the comments can be seen within a life course perspective with alcohol’s role changing: a sophisticated choice rather than a means to get intoxicated and lose control. During this life stage, women are becoming more aware of ageing, and life events (e.g. divorce, care responsibilities, grief, increased isolation) are influencing their drinking behaviour. The changing value of alcohol was occasionally made more explicit, usually around motives to reduce or stop drinking (‘*The [after] effects of even 2-3 small glasses of wine are just not worth it. I feel awful/I don’t sleep well—I eat junk—I get palpations & feel like I’m a waste of space the next day’, ‘Recovery time the next day from just one unit of alcohol takes a long time so it’s not really worth bothering to have an alcoholic drink’*).

#### Topic 1: Motives to drink

There were 156 open-text responses relating to other drinking motives (i.e. 16.6% of the sample provided further information). These were often expanding on motives included in the MDMS but occasionally identified unique drinking motives (see Table 6 for illustrative quotes).

**Table 6.** Motives to drink (a priori topic).

Theme	Subtheme 1	Subtheme 2	Quotes
Tool	Coping	Emotional and psychological states	• *To numb feelings* • *To cope with bad feelings* • *To relieve the sense of frustration and loneliness* • *Having a drink helps to alleviate the mundanity*
		Physical symptoms and pain relief	• *To forget how awful I feel and all my aches and pains* • *Numb pain* • *Pain relief when painkillers don’t work*
		Life events (significant and daily)	• *Drank more during divorce* • *Life seems more difficult* • *I’ve had an argument*
	Connection	Social	• *Make myself more sociable in the home* • *Sociable drink with partner in the evening* • *When I’m out at a social occasion*
		In memoriam	• *Drinking wine from the region my grandparents are from makes me feel connected* • *In memory of a late relative*
	Libido		• *It helps my libido* • *Due to lack of libido I sometimes have a drink to relax which helps with sex*
	Self-identity		• *I want people to think I’m good fun*
Taste and experience	Taste		• *I enjoy the taste of nice wine* • *I enjoy the taste and experience*
	Sophistication and discernment		• *I like the taste of well-made craft beer* • *I like the taste. . .it feels ‘grown-up’ and sophisticated* • *I drink more because I like the taste and less and less for the social or relaxation element*
	Environmental context		• *I like the taste, especially a little wine with food at Christmas* • *I like the taste. . . I enjoy a nice bottle of wine or a glass of whisky at home with my husband*
Routine	Habit		• *Habit* • *I’ve drank a bottle of wine every evening since I was 18 and certainly don’t wish to stop now*
	Me time		• *To mark the end of kid-time and start of grown-up time when the boys have gone to bed* • *A signifier of the end of the day/start of the evening and stopping ‘tasks’* • *After a long week at work*

*Tool:* Alcohol was often used as a tool, with participants trying to achieve something through drinking. By far the most dominant subtheme within tools was ‘*Coping*’. Respondents reported drinking to manage negative emotional and psychological states or self-medicate against physical pain. Alcohol was also used to manage stressful life events, whether these be significant (e.g. divorce) or daily stressors. Another subtheme was ‘*Connection’*, with respondents consuming alcohol to be sociable both inside and outside the home. As well as using alcohol to elevate the social experience, some participants were driven by a desire for social connection and this extended to seeking closeness with, and/or in memoriam of, those who have passed away. Occasionally women reported using alcohol to increase their ‘*libido*’, which is also likely driven by both a desire for connection and as a way to shape their identity (e.g. as a sexual woman). Other respondents referred to drinking as a way of portraying themselves as fun, again illustrating how alcohol is used to demonstrate preferred ‘*self-identity*’ which may facilitate connection.

*Taste and experience*: It was common for respondents to say they drank for the ‘*taste*’, although taste-based motives were often set within an ‘*environmental context*’ illustrating an enjoyable experience or memory (e.g. having a cold beer on a hot day). Linked with experience, were motives around trying new products which reflected ‘*sophistication and discernment*’ within respondent’s drinking behaviour, placing an importance on the wider experience of drinking, as opposed to focusing on intoxication effects. The comments within this theme suggest that taste-related motives are multifaceted, representing a complex interplay between emotions, relationships, and situations that are valued by the drinker and which have likely evolved over years of drinking.

*Routine*: For some, drinking had become a regular part of life, and something to signal different parts of the day or week. In this manner, the consumption of alcohol may be integrated into one’s everyday routine as a ‘*habit*’ but it can also be used purposefully to mark the passage of time and signal when women can step away from work and family commitments and have ‘*me time*’.

#### Topic 2: Motives to reduce drinking

One hundred and forty-nine responses (15.9%) related to reasons to reduce or stop drinking (see Table 7 for illustrative quotes).

**Table 7.** Motives to reduce drinking (a priori topic).

Theme	Subtheme	Quotes
Health and well-being	Health	• *Everything I read suggested that drinking alcohol at this time was unwise* • *Learned about how detrimental drinking was to my health* • *To reduce risk of breast cancer reoccurrence* • *I read it might increase the chance of dementia*
	Healthy ageing	• *Feel too old to keep up with the youngsters. . .Want to start being healthier. . .intention to change my routines and stop drinking*
	Concern	• *I decided to fully stop alcohol consumption. . .due to increasing volumes and it becoming a daily habit* • *I was becoming too reliant on alcohol—didn’t want this to lead to addiction* • *I had such problems with the perimenopause I became a raging alcoholic. . .I was completely on my knees because of it, I tried to deal with it myself for about a year before I finally realised I could not do it alone and needed serious help*
Tolerance and negative alcohol effects	Decreased tolerance	• *My tolerance has reduced i think in line with hormonal changes* • *Tolerance for alcohol is less*
	Hangovers	• *Unbearable hangovers* • *Hangovers are so much worse since menopause*
	Negative physical effects	• *Alcohol often causes a migraine* • *The little bit I did drink started to give me extremely bad tummies and headaches* • *Alcohol increased night sweats and palpitations* • *I can no longer drink alcohol as it makes me feel horrible*
	Sleep	• *Severely reduced drinking as couldn’t sleep and was doing everything I could to try to sleep* • *Too tired*
	Eating behaviour	• *To stop me snacking after wine*
	Inhibitory control	• *I also dislike the feeling of having less control when I have had a drink* • *Reduced inhibitions*
	Medication interactions	• *Interferes with the meds I take*
Coping	Mental health	• *To stop the anxiety* • *Alcohol definitely makes me feel worse and guilty*
	Perimenopausal symptoms	• *I cannot drink much any more as it makes anxiety worse which has become crippling since peri-menopause* • *Alcohol increased night sweats and palpitations* • *To stop the bloating* • *Cut out all alcohol as it exasperated the symptoms of peri & menopause* • *Alcohol makes me anxious, this has increased since peri-menopause started*
Financial		• *I can’t afford to. . .In a way, I am glad I can’t afford alcohol as I think I would use it more often if I did* • *To reduce unnecessary household expenditure after my husband was made redundant* • *No pension* • *Been going out less since lockdown/cost of living*

*Health and well-being*: A dominant theme, respondents showed awareness of alcohol’s potentially harmful effects on ‘*health*’, sometimes these comments related to a general desire to be healthy or to reduce the chances of specific illnesses (e.g. breast cancer). Additionally, there was an awareness that ‘*healthy ageing’* was becoming more important, which may be associated with some respondents showing significant ‘*concern*’ that their drinking was becoming an issue. Health concern comments often suggested respondents had chosen (or tried) abstinence rather than just reducing their intake.

*Tolerance and negative alcohol effects*: This theme is associated with health, but is focused on alcohol’s specific, acute effects. Many respondents reported a decrease in their ‘*tolerance’* to drinking alcohol. This fed directly into cutting down alcohol intake to avoid worsening ‘*hangovers*’ and other ‘*negative physical effects*’ (e.g. migraines, stomach issues). Other drinking deterrents were the effects of alcohol on ‘*sleep*’ and ‘*eating behaviour*’. Some respondents avoided alcohol due to its effects on ‘*inhibitory control’*, while others wanted to avoid negative ‘*medication interactions*’. Again, this shows a shift in what is important to women in mid-life, framing alcohol use as a sophisticated choice while wanting to avoid some of the loss of control aspects of drinking, which may not have been a consideration during earlier years (or, indeed, may be a desired alcohol effect).

*Coping*: Again, associated with health but these comments were placed within a coping framework. Just as some respondents reported coping-based drinking motives, others reported reducing their alcohol intake to cope with symptoms of ‘*mental ill-health*’. Comments often highlighted the belief that alcohol was making existing ‘*perimenopausal symptoms’* worse.

*Financial*: The cost of alcohol was identified as a prohibiting factor which was sometimes contextualised within personal and societal life events (e.g. redundancy, cost of living crisis).

#### Topic 3: Changing motives

One hundred and thirty-two responses (14.1%) related to how drinking motives changed since noticing menopausal symptoms (see Table 8 for illustrative quotes).

**Table 8.** How motives have changed since noticing symptoms of (peri)menopause (a priori topic).

Theme	Subtheme	Quotes
Coping		• *Drink more often because I feel I need a crutch*
	Mental health	• *Less to have fun, more to relieve stress* • *My anxiety has increased, its led to me wanting to drink more often to relax* • *More stress relief and low mood relief, used to be more celebratory* • *To relieve. . .depression*
	Physical health	• *To relieve pain* • *A bit more related to physical pain*
	Reward	• *Partner with cancer, setting up a start up business and a son with ADHD, life is much harder and I am shouldering much more of the burden alone, having a drink is my reward* • *‘Reward’ getting through the day*
	Perimenopause	• *I feel more anxious and brain foggy, it helps settle this sometimes but is worse the next day* • *To reduce the anxiety which I never felt before menopause*
	Isolation	• *Feel more down/low, don’t want to go out, rather stay in with a bottle wine*
Self-identity	Younger self	• *Trying to keep feeling young* • *To feel more confident and like the ‘old me’*
	Confidence	• *No confidence, feeling old and ugly* • *I don’t like myself. I’m over-the-hill job-wise, too old to look for a new relationship* • *More for false confidence and to fit in*
	HRT	• *I’ve started HRT and feel more like my old self (not so tired and anxious all the time). I find myself looking forward to having a drink. I’m trying to stop this urge*

*Coping*: The dominant theme, by far, was a shift towards negative reinforcing motives of drinking, including self-medication of ‘*mental and physical health symptoms*’. Pain management was common and often this change in motives was explicitly linked to emergence of ‘*perimenopausal symptoms*’. Occasionally, coping motives were framed as the respondent’s way to ‘*reward*’ themselves for dealing with stressful life situations. The use of alcohol as a means of coping with physical and mental health issues may reveal a lack of alternative treatment or support options, and that alcohol has been cemented as a sticking plaster for a plethora of issues in women’s lives. This is concerning as although some women talked about increased isolation, using alcohol to cope may also exacerbate this sense of isolation as women stay at home and drink to manage their negative states.

*Self-identity*: Some respondents were using alcohol to portray a preferred identity or to forget their current one. Respondents shared a very negative sense of becoming older which resulted in a lack of ‘*confidence*’, and the use of alcohol to retain a sense of the ‘*younger selves*’. Although ‘*HRT*’ was rarely mentioned, one person made the interesting observation that HRT had improved their perimenopausal symptoms and, with this, their enjoyment in drinking had returned and they felt like their old self again.

## Discussion

This study examined drinking behaviour across stages of menopause (pre, peri/meno, post), and whether factors associated with menopause (e.g. menopause symptoms, mental health) predicted alcohol use. As hypothesised, respondents who self-identified as perimenopausal had higher scores on menopause symptoms, negative affect (covering depression, anxiety, stress), lower scores on well-being, and were more likely to endorse negative reinforcing drinking motives. Both AUDIT score (indicative of hazardous/harmful drinking) and menopausal symptoms were positively associated with poorer mental health and loneliness, and negatively associated with well-being. Weekly unit consumption and AUDIT score were also positively associated with menopausal symptoms. As expected, negative reinforcement drinking motives were more strongly associated with AUDIT score and weekly consumption than positive reinforcement drinking motives. Negative drinking motives partially mediated the relationship between menopausal symptoms and alcohol use.

As expected from existing evidence, loneliness and lower subjective well-being predicted mental ill-health^41,42^ as did more severe psychosocial menopause symptoms.^43,44^ This indicates that mental health support during menopause should incorporate facilitation of social connection and self-efficacy which, potentially, will also have a positive impact on health behaviours (e.g. alcohol use).^45^

Although some studies suggest post-menopausal women experience depression more than pre- and/or perimenopausal women,^46,47^ we did not find that menopause stage predicted mental ill-health. However, the way perimenopause is understood, managed, and assessed is changing (e.g.^46^). These inconsistencies may weaken comparisons between studies. Although categorising stages of menopause can be a helpful descriptor (and we found some differences in our outcome measures between low risk and hazardous drinkers by menopause status) we would suggest the key focus in research should be the severity of menopausal symptoms.

Menopause symptoms (incl. total/domain scores) were positively associated with weekly alcohol use and hazardous drinking, as were measures of poorer mental health and well-being, and positive/negative reinforcement drinking motives. Consistent with other research,^48^ weekly consumption and hazardous/harmful drinking were more strongly associated with negative, relative to positive, reinforcement drinking motives. Additionally, the mediation analysis suggests that those with greater menopause symptoms and higher negative drinking motives may be at a greater risk of alcohol dependence and poorer mental health. Negative reinforcement covers behaviours aimed at reducing/removing something unwanted (e.g. loneliness/pain). This aligns with the self-medication hypothesis and motivational model of alcohol use and the large body of evidence highlighting negative reinforcement drinking motives as increasing risk of alcohol harms.^49–52^

Importantly, the association between negative reinforcement motives and alcohol harm is not just mediated by level of consumption.^53^ Therefore, harm can occur at lower drinking levels, which may be particularly relevant to populations in which current alcohol recommendations have not been tailored (e.g. mid/older aged women who are vulnerable to harm due to a range of biopsychosocial factors^2,20^). Although most respondents were categorised as low risk based on weekly consumption and AUDIT score, a meaningful proportion were categorised as hazardous/harmful drinkers (perimeno: 36.1%, premeno: 33.3%, postmeno: 23.2%). This underscores the need for more alcohol research in mid-older aged women.^54^

Menopausal symptom severity did not predict hazardous/harmful drinking or mental ill-health. However, negative (not positive) reinforcement drinking motives did mediate the relationship between menopausal symptoms and both hazardous/harmful drinking and mental ill-health (see Supplemental Table 3 for mental health outcomes). This suggests that some women with more severe menopause symptoms and who drink for negative reinforcement (e.g. coping-based reasons), might be at greater risk of hazardous/harmful drinking and poorer mental health. Again, this fits with the self-medication hypothesis and evidence that comorbidity between AUD and clinical mental ill-health conditions (e.g. depression, post-traumatic stress disorder [PTSD]) and psychosocial stress can be higher in women than men.^55,56^ Crucially, this new evidence can be exploited to better identify women at risk of hazardous/harmful drinking behaviours.

Our qualitative data reflected this finding, illustrating clear themes around using alcohol to cope with numerous stressors, including those highlighted in wider alcohol research on women (e.g. low mood, daily stress (e.g.^11,57^)). Additionally, stressors more common in midlife were highlighted, which fits with research showing increased stress and drinking during times of significant work and caregiving responsibilities, as well as shifting marital and caregiver relationships.^58,59^

Importantly, respondents reported drinking to cope with menopause-attributed stressors (e.g. sleep problems, pain, low mood), adding to a growing awareness that menopausal women use a variety of non-prescribed substances to manage various symptoms (e.g. joint/muscle discomfort, irritability, sleep problems, depression, anxiety, hot flashes).^60^ This concerning use of alcohol to manage pain etc. suggests this vulnerable group are not seeking and/or accessing appropriate treatment, something echoed in evidence that women are less likely than men to be prescribed pain medication, and report feeling mistrusted by healthcare professionals.^61,62^ Existing evidence identifies a lack of knowledge regarding menopausal symptoms (11.9% of our sample were unsure what stage of menopause they were in, although the typical age of this group indicated potential perimenopause), embarrassment, and negative experiences with healthcare professionals concerning perimenopause as barriers to seeking help.^63^ Together, this underscores an urgent need for better information for women and education/training for healthcare professionals and warrants careful design of support pathways to reduce substance (alcohol) related harm, which incorporates key issues, such as menopause.

Similar to research which finds that mental ill-health can be associated with decreased or increased alcohol use,^22,23^ our qualitative data found that negative mood and menopausal symptoms were a reason to reduce drinking in some respondents. This was often driven by a desire to be healthier, aligning with findings that women tend to become more health conscious as they age.^64^

Weight and sleep management were also frequently cited motives to reduce drinking, which fits with literature identifying these issues as common and some of the most bothersome menopausal symptoms.^5,65^ Sex hormones influence sleep, metabolic processes, and activity in women.^65–67^ Alcohol also affects latency to sleep and sleep quality,^68^ with women reporting greater sleepiness and disturbed sleep in response to intoxication, relative to men.^69,70^ Alcohol also influences eating behaviour and calorie intake.^71,72^ Importantly, research shows that women become more open to seeking and receiving information and support around illness prevention during midlife.^73,74^ We suggest midlife provides an ideal opportunity to engage with women regarding how healthier behaviours and coping skills can help them achieve their goals, and sleep and weight management could be targeted in these interventions.^75^

Adding richness to our findings, qualitative responses highlighted that alcohol serves multiple roles for women during menopausal stages: coping, shaping self-identity, enjoying life, and maintaining social connections.^54^ These roles are likely influenced by interactions between biological (hormonal, neuropharmacological), psychological (mental health, drinking motives/expectancies), lived experience and behaviour (trauma, drinking behaviour, alternative coping strategies).^76^ This ‘role-based’ perspective fits within value-based decision-making frameworks, which propose that the subjective value of alcohol can change across time and situation, based on a ‘dynamic integration process’ involving comparison of positive and negative consequences of drinking relative to consequences of alternative responses/behaviours.^77^ Public health interventions and treatments must recognise the complexity and gender-specific nature of alcohol use, and be flexible to women’s lived experiences and current needs. Interventions that fail to acknowledge the important roles alcohol plays during mid-older life may have limited effectiveness.^54^ In order to be inclusive to diverse groups of women, interventions must tackle the stigma associated with women’s alcohol use (often influenced by socioeconomic factors^78^) and provide practical and appropriate alternatives (which can therefore be valued higher than alcohol), rather than simply targeting a reduction in alcohol consumption.

### Limitations

Regarding limitations, we made efforts to recruit from more diverse backgrounds (e.g. White British 76.4% survey versus 81.7% in UK, Black 5% survey versus 4.2 in UK). However, more research is needed which is either representative of UK women or oversamples vulnerable and minority groups to ensure evidence-informed health policy and support is relevant across sociodemographic characteristics. Although our sample was sufficient for our analysis, as our research questions were exploratory, we did not conduct a power calculation. The study was not pre-registered, which may limit the transparency of our analytical approach and increase the potential for bias in the interpretation of results. Future research should conduct a priori power analysis appropriate for their design and recruit larger cohorts to allow longitudinal modelling of drinking trajectories across stages of menopause. This will avoid limitations around cross-sectional mediation analysis in identifying cause-and-effect relationships. Studies should track how medications used to manage menopause may influence drinking over time. For instance, although few respondents referred to HRT, there was some suggestion that HRT’s success at reducing menopausal symptoms resulted in a return to alcohol use due to improved mood/well-being. Similarly, although we received a large number of qualitative responses and findings reflected existing literature, there are clearly complex, bi-directional associations between alcohol use, menopause, and well-being. Future research should include appropriate qualitative methods to capture women’s experiences in more depth.

Another potential limitation is the assessment tools used. We used a slightly reduced cut-off AUDIT score of 7 to indicate hazardous drinking, as suggested for female drinkers,^27^ yet this is still conservative. A UK accuracy report found a score of 4 in women was optimal for identifying hazardous drinking in 18–35 years olds.^28^ Reporting drinking behaviour by this lower gender-based cut-off score, the proportion of hazardous-dependent drinkers in our sample rose from 30.9% to 52% (see Table 1). We therefore call for more research to validate research and clinical assessment tools in mid-older women, a time in which biological changes may increase risk of alcohol harm.^20,21^ It is important to note that as some of the recruitment was via social media, this may bias the sample towards those experiencing a greater level of symptoms. We also did not ask about hormonal contraception, and recommend that this is measured in future research. Finally, participants self-reported their menopause status. While women’s experience of symptoms is paramount (NICE guidelines recommend hormone assessment only be considered in female patients under 45 years of age^79^), using objective measures of hormone levels could be used in future studies to examine the relationship between alcohol use and biological processes across menopause status. However, this would require multiple hormonal assessments, to determine both absolute and fluctuating hormonal levels. While age is often used as a proxy for menopausal status, it is an unreliable predictor due to significant individual variability in the timing and experience of menopause, as well as the high proportion of women who report uncertainty about their status.^80^ As with any study relying on self-report, the accuracy of the data is subject to biases, including recall bias and social desirability bias, which may have influenced participants’ responses. However, data was anonymous which reduces any potential issues around lack of self-disclosure of sensitive information (e.g. alcohol use, menopause status).^81^

## Conclusions

This study is the first in the UK to evidence the associations between menopausal symptoms, mental health, and alcohol use. Some women who are experiencing the negative symptoms of menopause and endorse negative reinforcing drinking motives may be at heightened risk of heavier alcohol use. Given the increased vulnerability to alcohol harms during mid-older life stages, there is a clear need for alcohol interventions and treatments to incorporate therapeutic components targeting drivers of drinking during menopause. This is important both from an individual and societal perspective. The peri-post menopausal stage of life is the longest life stage for many women; maximising good health and well-being is likely to have significant health and societal cost benefits.

## Supplemental Material

sj-docx-1-whe-10.1177_17455057251359767 – Supplemental material for Women’s alcohol use in mid-life: Identifying associations between menopause symptoms, drinking behaviour, and mental health Supplemental material, sj-docx-1-whe-10.1177_17455057251359767 for Women’s alcohol use in mid-life: Identifying associations between menopause symptoms, drinking behaviour, and mental health by Emma L. Davies, Sam Burton, Rebecca Monk, Elena Murdoch, Eiluned Pearce and Abigail K. Rose in Women's Health

sj-docx-2-whe-10.1177_17455057251359767 – Supplemental material for Women’s alcohol use in mid-life: Identifying associations between menopause symptoms, drinking behaviour, and mental health Supplemental material, sj-docx-2-whe-10.1177_17455057251359767 for Women’s alcohol use in mid-life: Identifying associations between menopause symptoms, drinking behaviour, and mental health by Emma L. Davies, Sam Burton, Rebecca Monk, Elena Murdoch, Eiluned Pearce and Abigail K. Rose in Women's Health

sj-docx-3-whe-10.1177_17455057251359767 – Supplemental material for Women’s alcohol use in mid-life: Identifying associations between menopause symptoms, drinking behaviour, and mental health Supplemental material, sj-docx-3-whe-10.1177_17455057251359767 for Women’s alcohol use in mid-life: Identifying associations between menopause symptoms, drinking behaviour, and mental health by Emma L. Davies, Sam Burton, Rebecca Monk, Elena Murdoch, Eiluned Pearce and Abigail K. Rose in Women's Health

sj-docx-4-whe-10.1177_17455057251359767 – Supplemental material for Women’s alcohol use in mid-life: Identifying associations between menopause symptoms, drinking behaviour, and mental health Supplemental material, sj-docx-4-whe-10.1177_17455057251359767 for Women’s alcohol use in mid-life: Identifying associations between menopause symptoms, drinking behaviour, and mental health by Emma L. Davies, Sam Burton, Rebecca Monk, Elena Murdoch, Eiluned Pearce and Abigail K. Rose in Women's Health

sj-docx-5-whe-10.1177_17455057251359767 – Supplemental material for Women’s alcohol use in mid-life: Identifying associations between menopause symptoms, drinking behaviour, and mental health Supplemental material, sj-docx-5-whe-10.1177_17455057251359767 for Women’s alcohol use in mid-life: Identifying associations between menopause symptoms, drinking behaviour, and mental health by Emma L. Davies, Sam Burton, Rebecca Monk, Elena Murdoch, Eiluned Pearce and Abigail K. Rose in Women's Health

sj-docx-6-whe-10.1177_17455057251359767 – Supplemental material for Women’s alcohol use in mid-life: Identifying associations between menopause symptoms, drinking behaviour, and mental health Supplemental material, sj-docx-6-whe-10.1177_17455057251359767 for Women’s alcohol use in mid-life: Identifying associations between menopause symptoms, drinking behaviour, and mental health by Emma L. Davies, Sam Burton, Rebecca Monk, Elena Murdoch, Eiluned Pearce and Abigail K. Rose in Women's Health

sj-docx-7-whe-10.1177_17455057251359767 – Supplemental material for Women’s alcohol use in mid-life: Identifying associations between menopause symptoms, drinking behaviour, and mental health Supplemental material, sj-docx-7-whe-10.1177_17455057251359767 for Women’s alcohol use in mid-life: Identifying associations between menopause symptoms, drinking behaviour, and mental health by Emma L. Davies, Sam Burton, Rebecca Monk, Elena Murdoch, Eiluned Pearce and Abigail K. Rose in Women's Health

sj-docx-8-whe-10.1177_17455057251359767 – Supplemental material for Women’s alcohol use in mid-life: Identifying associations between menopause symptoms, drinking behaviour, and mental health Supplemental material, sj-docx-8-whe-10.1177_17455057251359767 for Women’s alcohol use in mid-life: Identifying associations between menopause symptoms, drinking behaviour, and mental health by Emma L. Davies, Sam Burton, Rebecca Monk, Elena Murdoch, Eiluned Pearce and Abigail K. Rose in Women's Health
