# Supplementary material for: Women’s alcohol use in mid-life: Identifying associations between menopause symptoms, drinking behaviour, and mental health
Source: Womens Health (Lond). 2025 Oct 8;21:17455057251359767. doi: 10.1177/17455057251359767 (PMC12511719; doi:10.1177/17455057251359767)
Supplement: sj-docx-2-whe-10.1177_17455057251359767 – Supplemental material for Women’s alcohol use in mid-life: Identifying associations between menopause symptoms, drinking behaviour, and mental health [file sj-docx-2-whe-10.1177_17455057251359767.docx]

**Survey: Alcohol use, menopause symptoms and wellbeing**

**Demographic information**
We are asking for this information because alcohol use, health and wellbeing may be influenced by individual characteristics. So, we need to take these into account when looking at the survey data. Please highlight your responses by clicking on the relevant option. You can add extra information in the text boxes, where applicable.

1. What is your age?

|  | 40 | 42 | 44 | 46 | 48 | 50 | 53 | 55 | 57 | 59 | 61 | 63 | 65 |
| --- | --- | --- | --- | --- | --- | --- | --- | --- | --- | --- | --- | --- | --- |

| 1 () | 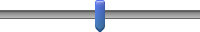 |
| --- | --- |

1. Ethnicity

- Asian - British
- Asian - Other ______________
- Black - British
- Black - Other ______________
- Mixed - Any _______________
- White British
- White - Other ______________
- Any other ethnic origin group___________
- I prefer not to answer this question

1. Relationship status

- Married or co-habitating
- Single
- In a relationship (not co-habitating)
- Divorced or separated
- Widowed
- I prefer not to answer this question

1. What is the highest level of education you have completed?

- Primary school
- Secondary school (e.g. GCSE)
- Secondary School / College (e.g. A'Level)
- Trade/technical/vocational training
- Bachelor's degree
- Master's degree
- Doctorate degree
- Professional degree
- Other, please specify______________
- I prefer not to answer this question

1. What is your current occupation?

- Maternity leave
- Employed, full time
- Employed, part time
- Self-employed
- Retired
- Stay at home mum / homemaker
- Unemployed
- Unable to work
- I prefer not to answer this question

1. What is your average household income (before tax)?

|  | 0 | 20 | 40 | 60 | 80 | 100 | 120 | 140 | 160 | 180 | 200 |
| --- | --- | --- | --- | --- | --- | --- | --- | --- | --- | --- | --- |

| Thousand £ p/year () | 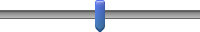 |
| --- | --- |

8. In which country do you currently live?

▼ Afghanistan (1) ... Zimbabwe (1357)

Display This Question:

If List of Countries = United Kingdom of Great Britain and Northern Ireland

1. Where in the UK do you live?

- Northern Ireland
- Scotland
- Wales
- England, London
- England, North West
- England, North East
- England, Yorkshire and Humber
- England, West Midlands
- England, East Midlands
- England, South West
- England, South East
- England, East Anglia
- I prefer not to answer this question

1. Sexuality

- Heterosexual
- Lesbian/Gay
- Bisexual
- Other, please specify __________________________________________________
- I prefer not to answer this question

1. What gender do you identify as?

- Female
- Transman
- Non-binary
- Gender fluid
- Other __________________________________________________
- I prefer not to answer this question

1. How many children do you have?

|  | 0 | 1 | 2 | 3 | 4 | 5 | 6 | 7 | 8 | 9 | 10 | 11 | 12 | 13 | 14 | 15 |
| --- | --- | --- | --- | --- | --- | --- | --- | --- | --- | --- | --- | --- | --- | --- | --- | --- |

| Number of children () | 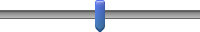 |
| --- | --- |

1. In the text space, please enter year of birth (please enter child 1 as the oldest, e.g., 1: 2010, 2: 2016)

- 1 __________________________________________________
- 2 __________________________________________________
- 3 __________________________________________________
- 4 __________________________________________________
- 5 __________________________________________________
- 6 __________________________________________________
- 7 __________________________________________________
- If you have more than 8 children, please enter the birth year of the youngest (9) __________________________________________________

1. Please provide the date for the first day of your last period (an approximate date is fine)

*Calendar tool was provided.*

1. Have you had a partial or full hysterectomy?

- Yes
- No
- Prefer not to answer this question

1. Regardless of whether you have spoken to a health professional, do you feel you are 1) premenopausal, 2) perimenopausal or menopausal, or 3) post-menopausal?

   We have combined perimenopausal and menopausal, as we can only be certain we have been menopausal, after we have not had a period for 12 months. By this point we are postmenopausal.

|  | No | Yes, I do not take any medication for menopausal symptoms (e.g. HRT) | Yes, I take medication for menopausal symptoms (e.g., HRT) | Not sure |
| --- | --- | --- | --- | --- |
| **Premenopausal** (usually before your early-mid 40s). You are menstruating in a way that is typical to you. You have not noticed any changes which may be due to perimenopause (outlined below). |  |  |  |  |
| **Perimenopausal** (often starts early-mid 40s) or **menopausal** (often starts early 50s). Symptoms can include: irregular and/or reduced periods, hot flushes, night sweats, sleep disturbances, cognitive problems [brain fog], mood changes, reduced libido [sex drive], vaginal dryness/discomfort etc |  |  |  |  |
| **Postmenopausal**. Once you have not had a period for 12 months you are considered postmenopausal. You may experience a variety of symptoms, including those listed above under perimenopause.) |  |  |  |  |

1. Please tell us if you are currently taking any treatments to manage symptoms associated with stages of menopause (click all that apply).

- Oestrogen
- Progesterone
- Testosterone
- Clonidine
- Gabapentin
- Antidepressants
- Other_________

1. If applicable, at what age did you start noticing symptoms of (peri)menopause?

|  | 10 | 15 | 20 | 25 | 30 | 35 | 40 | 45 | 50 | 55 | 60 | 65 |
| --- | --- | --- | --- | --- | --- | --- | --- | --- | --- | --- | --- | --- |

|  | 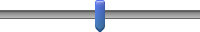 |
| --- | --- |

1. If known, what is the cause of your (peri/post)menopausal symptoms?

- Natural
- Cancer
- Gynaecological surgery (e.g., full/partial hysterectomy)
- Other ___________________________

1. *The Menopause-Specific Quality of Life Questionnaire (MENQOL) was included here to assess presence and impact of menopause symptoms (29 items).*

This section asks about your past alcohol use. We are asking for this information because we want to identify things that might be associated with (and without) alcohol use during stages of menopause, and historical drinking maybe important.

These questions are asked with compassion and without judgement. Your data is anonymous, please answer as truthfully as you can.

1. Have you ever sought treatment to reduce your drinking or been diagnosed with an alcohol use disorder?

|  | Yes (1) | No (2) | I prefer not to answer this question (3) |
| --- | --- | --- | --- |
| Before noticing symptoms of (peri)menopause (1) |  |  |  |
| Since noticing symptoms of (peri)menopause (2) |  |  |  |

1. BEFORE you noticed any symptoms associated with stages of (peri)menopause, approximately how many of the following drinks did you drink a week?

   If you are premenopausal, please skip this question.

|  | 0 | 2 | 4 | 6 | 8 | 10 | 12 | 14 | 16 | 18 | 20 | 22 | 24 | 26 | 28 | 30 |
| --- | --- | --- | --- | --- | --- | --- | --- | --- | --- | --- | --- | --- | --- | --- | --- | --- |

| Standard glass of wine (175 ml) () | 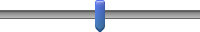 |
| --- | --- |
| Large glass of wine (250 ml) () | 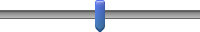 |
| Bottle of wine () | 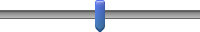 |
| Pint of lager/beer/ale/cider () | 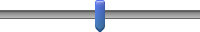 |
| Half pint (or bottle) of lager/beer/ale/cider () | 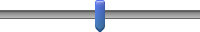 |
| Shots (35ml) of spirits (with or without mixer) () | 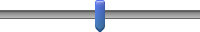 |
| Bottles of alcopops () | 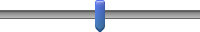 |

1. *Timeline Followback (TLFB) was included here, to assess weekly alcohol use using a 2-week diary format*
2. *The Alcohol Use Disorders Identification Test (AUDIT) was included here to assess alcohol use and potentially harmful drinking behaviour (10 items).*

|  |
| --- |

1. *The Maternal Drinking Motive scale (MDMS) was included here to assess positive and negative reinforcing drinking motives*
2. Are there any other reasons you drink alcohol?
   Please use the box below to list these.
3. Do you think your **reasons** for drinking have changed since you have noticed symptoms associated with the stages of menopause?

- Yes, please specify _________________________________
- No
- Unsure

1. What planet do you live on?
   This is an attention check

- Neptune
- Earth
- Jupiter
- Venus

1. Reasons to reduce/stop drinking
    
   Since noticing symptoms associated with the stages of menopause, have you stopped or reduced your alcohol consumption for a specific reason?
   Please tick all that apply

- No, I have not stopped or reduced my drinking
- Yes, I'm too tired to drink alcohol
- Yes, drinking disrupts my sleep
- Yes, I want to be healthy
- Yes, I want to set a good example for my child(ren)
- Yes, I don't want to risk harming my child(ren)
- Yes, to help manage my weight
- Yes, I feel guilt about drinking
- Yes, but not for any specific reason
- Yes, to help manage cognitive symptoms (e.g., brain fog)
- Yes, to help manage feelings of anxiety
- Yes, to help manage feelings of low mood
- Yes, to help manage low libido (sex drive)
- Yes, but for another reason. Please specify___________
- Don't know

There are important links between alcohol use and well-being, but not much research looks at these links in women who may be experiencing stages of menopause. This section asks you to complete validated measures on mental health and wellbeing.

1. *The Depression, Anxiety and Stress Scale was included here to assess negative affect (anxiety, depression, stress). A mean score for each subscale was calculated.*
2. *The UCLA Loneliness Scale was included here to assess loneliness over 3 items.*
3. *The World Health Organisation’s Wellbeing Scale (WHO-5) was included here to assess current wellbeing.*
