# Supplementary material for: Women’s alcohol use in mid-life: Identifying associations between menopause symptoms, drinking behaviour, and mental health
Source: Womens Health (Lond). 2025 Oct 8;21:17455057251359767. doi: 10.1177/17455057251359767 (PMC12511719; doi:10.1177/17455057251359767)
Supplement: sj-docx-3-whe-10.1177_17455057251359767 – Supplemental material for Women’s alcohol use in mid-life: Identifying associations between menopause symptoms, drinking behaviour, and mental health [file sj-docx-3-whe-10.1177_17455057251359767.docx]

Indirect effect of negative motives

*b* = .07, 95% CI [.02, .14]

Total effect

Positive motives

Direct effect

*b* = 1.97, *p*=.001

*b* = -.15, *p*=.001

*b* = .12, *p*<.001

*b* = .059, *p*=.009

DASS

Negative motives

MENQOL

*b* = .08, 95% CI [.02, .15]

*b* = -.06, *p*=.616

Indirect effect of positive motives

*b* = .01, 95% CI [-.03, .05]

***Figure 1:*** Multiple mediation model of the relationship between MENQOL and DASS, mediated by negative and positive drinking motives. Co-variates in the model are AUDIT, menopause status and HRT status. The confidence interval for the indirect effect is a BCa bootstrapped CI based on 5000 samples, *R^2^* = .306, p<.001.
