# Supplementary material for: Women’s alcohol use in mid-life: Identifying associations between menopause symptoms, drinking behaviour, and mental health
Source: Womens Health (Lond). 2025 Oct 8;21:17455057251359767. doi: 10.1177/17455057251359767 (PMC12511719; doi:10.1177/17455057251359767)
Supplement: sj-docx-4-whe-10.1177_17455057251359767 – Supplemental material for Women’s alcohol use in mid-life: Identifying associations between menopause symptoms, drinking behaviour, and mental health [file sj-docx-4-whe-10.1177_17455057251359767.docx]

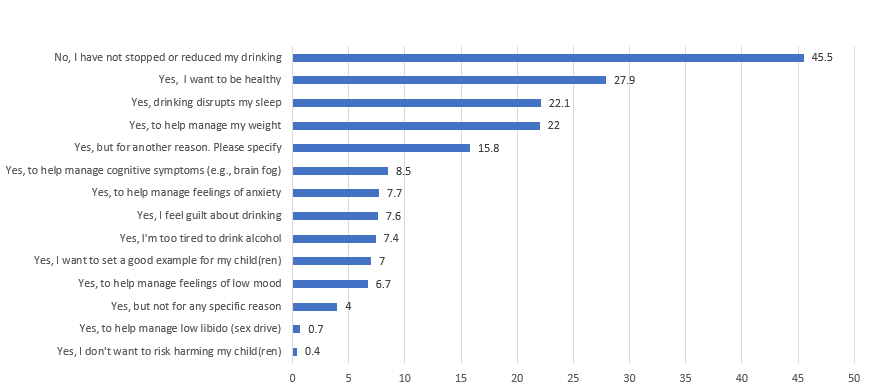


**Figure 2**: Since noticing symptoms associated with the stages of menopause, have you stopped or reduced your alcohol consumption for a specific reason? Data included for people identifying as peri- or post-menopausal only (N=673)
