# Supplementary material for: Women’s alcohol use in mid-life: Identifying associations between menopause symptoms, drinking behaviour, and mental health
Source: Womens Health (Lond). 2025 Oct 8;21:17455057251359767. doi: 10.1177/17455057251359767 (PMC12511719; doi:10.1177/17455057251359767)
Supplement: sj-docx-5-whe-10.1177_17455057251359767 – Supplemental material for Women’s alcohol use in mid-life: Identifying associations between menopause symptoms, drinking behaviour, and mental health [file sj-docx-5-whe-10.1177_17455057251359767.docx]

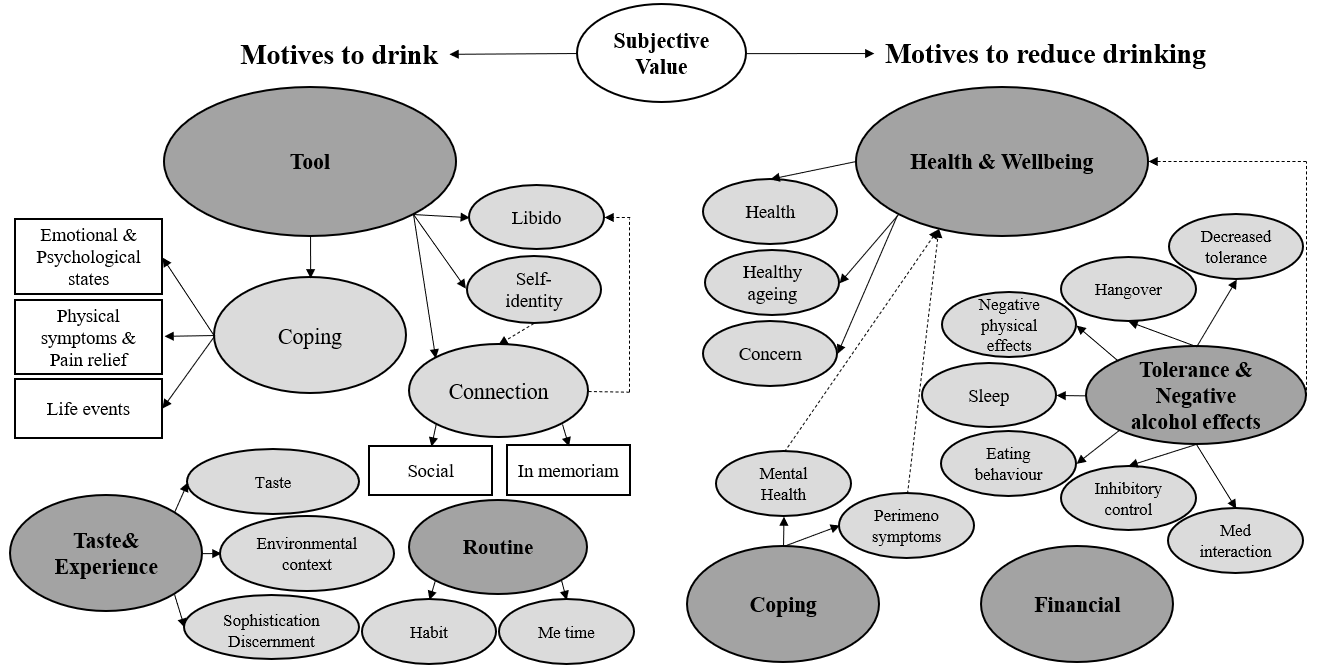


**Figure 3**. Schematic of qualitive themes. Women reported a range of motives to drink and to reduce drinking. Coping was a dominant theme across all *a priori* topics. Alcohol was used to manage negative physical and mental health symptoms. Although women reported reducing or stopping drinking to cope, the primary reasons for changing motives (since noticing perimenopausal symptoms) identified a shift from drinking for social reasons to drinking to cope both with health-related symptoms and life events. This highlights that (peri)menopause may be a stage of life where women are at risk of using alcohol either to self-medicate against various unwanted issues (e.g. mental/physical health symptoms, isolation) or to reward themselves for dealing with these issues. There was an awareness of ageing and how drinking behaviours could be detrimental but also used to shape self-identity. Women’s desire to be healthy highlights opportunities for health promotion, but the reported use of alcohol as a coping mechanism and pain reliever suggests opportunities are being missed.

Legend: Dark grey: Overarching themes. Light grey: Subthemes, level 1. White, rectangle: Subthemes, level 2
