# Supplementary material for: Women’s alcohol use in mid-life: Identifying associations between menopause symptoms, drinking behaviour, and mental health
Source: Womens Health (Lond). 2025 Oct 8;21:17455057251359767. doi: 10.1177/17455057251359767 (PMC12511719; doi:10.1177/17455057251359767)
Supplement: sj-docx-6-whe-10.1177_17455057251359767 – Supplemental material for Women’s alcohol use in mid-life: Identifying associations between menopause symptoms, drinking behaviour, and mental health [file sj-docx-6-whe-10.1177_17455057251359767.docx]

**Supplementary table 1 :** Comparison of measures by HRT status

|  | Whole sample | Not on HRT | HRT | p value * |
| --- | --- | --- | --- | --- |
| **Drinker status N=934** |  |  |  | .813 |
| Non-drinker N(%) | 107 (11.5) | 79 (11.3) | 28 (12.0) |  |
| Current drinker N(%) | 827 (88.5) | 623 (88.7) | 206 (88.0) |  |
| **Ethnicity** |  |  |  | .015 |
| Asian | 45 (4.8) | 41 (5.8) | 4 (1.7) |  |
| Black | 51 (5.4) | 45 (6.4) | 6 (2.6) |  |
| Mixed | 55 (5.9) | 41 (5.8) | 14 (6.0) |  |
| White | 776 (82.9) | 568 (80.9) | 208 (88.9) |  |
| Other | 9 (1.0) | 7 (1.0) | 2 (0.9) |  |
| **Education** |  |  |  | .001 |
| Secondary or below | 345 (37.9) | 264 (37.7) | 90 (38.5) |  |
| Degree | 374 (40.0) | 300 (42.8) | 74 (31.6) |  |
| Postgraduate | 207 (22.1) | 137 (19.5) | 70 (29.9) |  |
| **AUDIT categories – drinkers only** |  |  |  | .072 |
| Low risk N(%) | 571 (69.0) | 443 (71.3) | 128 (62.1) |  |
| Increasing risk N(%) | 196 (23.7) | 139 (22.4) | 57 (27.7) |  |
| Higher risk N(%) | 29 (3.5) | 19 (3.1) | 10 (4.9) |  |
| Possible dependence N(%) | 31 (3.7) | 20 (3.2) | 11 (5.3) |  |
| **Alcohol consumption** |  |  |  |  |
| Units pre menopause M (SD) N=821 | 18.49 (25.23) | 17.37 (25.37) | 21.68 (24.57) | .031 |
| Units pre menopause Mdn (IQR) | 10 (22.23) | 9.45 (20.68) | 15.8 (24.4) |  |
| Current weekly units M(SD) | 13.51 (17.88) | 12.57 (16.39) | 16.38 (21.57) | .007 |
| Current weekly units Mdn (IQR) | 7.45 (16.39) | 6.75 (159.6) | 9.53 (152.4) |  |
| 0-14 units N (%) | 556 (60.8) | 425 (70.5) | 129 (63.5) | .082 |
| 15-34 units N (%) | 177 (19.3) | 129 (21.4) | 48 (23.6) |  |
| 35 units and above N (%) | 75 (8.2) | 49 (8.1) | 26 (12.8) |  |
| AUDIT score M (SD) | 6.37 (5.33) | 6.04 (5.06) | 7.34 (5.97) | .002 |
| AUDIT score Mdn (IQR) | 5 (6) | 5 (6) | 5 (7) |  |
| AUDIT-C M (SD) | 4.52 (2.71) | 4.41 (2.65) | 4.84 (2.88) | .043 |
| AUDIT-C Mdn (IQR) | 4 (4) | 4 (4) | 4 (3.5) |  |
| **Motives** |  |  |  |  |
| Negative motives | 18.37 (8.71) | 17.56 (8.14) | 20.80 (9.85) | <.001 |
| Positive motives | 14.12 (4.19) | 14.00 (4.26) | 14.48 (3.98) | .157 |
|  |  |  |  |  |
| **Whole sample** |  |  |  |  |
| Mental health M (SD) |  |  |  |  |
| MENQOL total | 2.48 (1.29) | 2.32 (1.27) | 2.94 (1.25) | <.001 |
| MENQOL Vasomotor | 1.77 (1.73) | 1.69 (1.69) | 2.02 (1.75) | .012 |
| MENQOL Psychosocial | 2.53 (1.63) | 2.33 (1.58) | 3.12 (1.64) | <.001 |
| MENQOL Physical | 2.61 (1.36) | 2.47 (1.35) | 3.03 (1.29) | <.001 |
| MENQOL Sexual | 2.35 (1.86) | 2.16 (1.79) | 2.93 (1.98) | <.001 |
| Loneliness | 5.03 (2.00) | 4.99 (2.00) | 5.15 (2.01) | .284 |
| DASS | 5.59 (5.54) | 5.28 (5.50) | 6.52 (5.55) | .003 |
| WHO5 | 44.90 (22.65) | 46.30 (22.96) | 40.67 (21.19) | .001 |
