# Supplementary material for: Women’s alcohol use in mid-life: Identifying associations between menopause symptoms, drinking behaviour, and mental health
Source: Womens Health (Lond). 2025 Oct 8;21:17455057251359767. doi: 10.1177/17455057251359767 (PMC12511719; doi:10.1177/17455057251359767)
Supplement: sj-docx-7-whe-10.1177_17455057251359767 – Supplemental material for Women’s alcohol use in mid-life: Identifying associations between menopause symptoms, drinking behaviour, and mental health [file sj-docx-7-whe-10.1177_17455057251359767.docx]

**Supplementary Table 2**: ANCOVA results showing differences between low risk (AUDIT score <8) and hazardous and higher risk drinkers (AUDIT ≥8) on MENQOL, negative affect and drinking motives for respondents at each stage of the menopause, controlling for HRT.

|  | Whole sample | | Pre-menopausal | | Peri-menopausal | | Post-menopausal | |
| --- | --- | --- | --- | --- | --- | --- | --- | --- |
|  | Low risk  N=571 | Hazardous  N=256 | Low risk  N=66 | Hazardous  N=30 | Low risk  N=223 | Hazardous  N=126 | Low risk  N=216 | Hazardous  N=65 |
| MENQOL | 2.34 (1.28) | 2.74 (1.27) | **1.47 (1.15)** | **2.39 (1.26) *** | 2.60 (1.19) | 2.88 (1.24) | 2.41 (1.31) | 2.68 (1.18) |
| MENQOL vasomotor | 1.66 (1.69) | 2.02 (1.73) | 0.91 (1.47) | 1.80 (1.82) | 1.88 (1.58) | 2.21 (1.75) | 1.80 (1.89) | 1.90 (1.72) |
| MENQOL psychosocial | 2.32 (1.56) | 2.98 (1.65) | **1.62 (1.33)** | **2.68 (1.66) *** | **2.68 (1.59)** | **3.26 (1.57)*** | 2.21 (1.55) | 2.58 (1.59) |
| MENQOL physical | 2.49 (1.35) | 2.82 (1.36) | **1.57 (1.20)** | **2.45 (1.29) *** | 2.73 (1.29) | 2.92 (1.32) | 2.55 (1.36) | 2.83 (1.32) |
| MENQOL sexual | 2.32 (1.89) | 2.52 (1.82) | 1.13 (1.40) | 2.03 (1.88) | 2.44 (1.81) | 2.50 (1.78) | 2.68 (1.97) | 2.85 (1.85) |
|  |  |  |  |  |  |  |  |  |
| Loneliness | 4.85 (1.95) | 5.38 (2.11) | 5.27 (2.29) | 4.88 (1.87) | **4.83 (1.95)** | **5.63 (2.08)**** | 4.68 (1.81) | 5.12 (2.13) |
| DASS | 4.62 (4.97) | 7.14 (5.77) | 3.92 (4,41) | 5.90 (4.47) | **5.27 (5.53)** | **7.99 (6.01)**** | 4.14 (4.52) | 5.94 (5.67) |
| WHO-5 | 47.84 (22.25) | 39.78 (22.17) | 55.70 (19.87) | 42.13 (18.26) | **44.79 (22.16)** | **36.86 (21.04)*** | 49.35 (22.43) | 45.72 (23.74) |
|  |  |  |  |  |  |  |  |  |
| Negative motives | 16.02 (8.42) | 23.52 (6.95) | **14.20 (6.48)** | **21.07 (6.20)**** | **16.69 (8.98)** | **23.76 (6.96)**** | **15.95 (8.36)** | **23.71 (7.33)**** |
| Positive motives | 13.38 (4.12) | 15.74 (3.89) | 13.74 (4.54) | 15.63 (3.77) | **13.39 (3.99)** | **15.46 (3.63)**** | **13.01 (4.25)** | **15.94 (4.30)**** |
|  |  |  |  |  |  |  |  |  |

Note * indicates where one way ANCOVA for difference between low risk and hazardous drinkers controlling for HRT status p<05. ** p ≤.001
