# Supplementary material for: Women’s alcohol use in mid-life: Identifying associations between menopause symptoms, drinking behaviour, and mental health
Source: Womens Health (Lond). 2025 Oct 8;21:17455057251359767. doi: 10.1177/17455057251359767 (PMC12511719; doi:10.1177/17455057251359767)
Supplement: sj-docx-8-whe-10.1177_17455057251359767 – Supplemental material for Women’s alcohol use in mid-life: Identifying associations between menopause symptoms, drinking behaviour, and mental health [file sj-docx-8-whe-10.1177_17455057251359767.docx]

Due to the known associations between mental health and alcohol use, we also looked at whether menopause outcomes predicted mental health in the sample. Menopause and HRT status were not significant predictors in the model. MENQOL’s psychosocial domain, loneliness and WHO-were significant predictors of DASS.

**Supplementary Table 3:** Negative binomial regression model showing variables associated with DASS scores for participants in the sample.

|  |  |  |  |  |  | 95% CI |  |
| --- | --- | --- | --- | --- | --- | --- | --- |
|  | B | Std error | Wald Chi Sq | Sig. | IRR | Lower | Upper |
| Intercept | 1.57 | 0.285 | 30.377 | <.001 | 4.809 | 2.751 | 8.406 |
|  |  |  |  |  |  |  |  |
| **Menopause** |  |  |  |  |  |  |  |
| Not sure | -0.293 | 0.158 | 3.46 | 0.063 | 0.746 | 0.548 | 1.016 |
| Post-meno | -0.147 | 0.134 | 1.215 | 0.270 | 0.863 | 0.664 | 1.121 |
| Peri-meno | -0.192 | 0.128 | 2.246 | 0.134 | 0.825 | 0.642 | 1.061 |
|  |  |  |  |  |  |  |  |
| **HRT** |  |  |  |  |  |  |  |
| Using HRT | -0.023 | 0.093 | 0.06 | 0.807 | 0.978 | 0.815 | 1.173 |
| E**ducation** |  |  |  |  | 1 |  |  |
| Postgraduate | 0.097 | 0.113 | 0.741 | 0.389 | 1.102 | 0.883 | 1.376 |
| Degree | 0.046 | 0.092 | 0.256 | 0.613 | 1.047 | 0.875 | 1.254 |
|  |  |  |  |  |  |  |  |
| **Ethnicity** |  |  |  |  |  |  |  |
| Other | -0.054 | 0.436 | 0.015 | 0.902 | 0.948 | 0.403 | 2.226 |
| White | -0.17 | 0.172 | 0.981 | 0.322 | 0.844 | 0.603 | 1.181 |
| Mixed | 0.052 | 0.229 | 0.051 | 0.821 | 1.053 | 0.673 | 1.649 |
| Black | -0.299 | 0.235 | 1.611 | 0.204 | 0.742 | 0.468 | 1.176 |
|  |  |  |  |  |  |  |  |
| **MENQOL** |  |  |  |  |  |  |  |
| Vasomotor | -0.034 | 0.027 | 1.608 | 0.205 | 0.966 | 0.917 | 1.019 |
| Psychosocial | **0.275** | **0.041** | **45.768** | **<.001** | **1.316** | **1.215** | **1.425** |
| Physical | -0.002 | 0.048 | 0.002 | 0.964 | 0.998 | 0.909 | 1.096 |
| Sexual | -0.017 | 0.025 | 0.434 | 0.510 | 0.984 | 0.936 | 1.033 |
|  |  |  |  |  |  |  |  |
| **Mental health** |  |  |  |  |  |  |  |
| Loneliness | **0.084** | **0.021** | **15.693** | **<.001** | **1.088** | **1.044** | **1.134** |
| WHO5 | **-0.02** | **0.002** | **84.879** | **<.001** | **0.98** | **0.976** | **0.984** |
|  |  |  |  |  |  |  |  |
| **Income** | 0 | 0.001 | 0.021 | 0.885 | 1 | 0.998 | 1.002 |

Notes: N = 898; Probability distribution = normal; link function = identity; Goodness of fit value/df = 13.40; Omnibus test *χ2* = 740.40 (20), p<.001.
